# Chemotherapy-related adverse drug reaction and associated factors among adult cancer patient attending Jimma medical center oncology unit, Southwest Ethiopia

**DOI:** 10.1371/journal.pone.0321785

**Published:** 2025-05-16

**Authors:** Mukerem Sultan Shrmeka, Mubarik Fetu Semman, Biruk Tafese Moges, Feki Nekir Dereja, Aster Wakjira Garedo

**Affiliations:** 1 Department of Pharmacy, College of Medicine and Health Science, Wolkite University, Wolkite, Ethiopia; 2 Department of Clinical Pharmacy, School of Pharmacy, College of Medicine and Health Science, Jimma University, Jimma, Ethiopia; Fujian Provincial Hospital, CHINA

## Abstract

**Background:**

In 2017, reports of adverse drug reactions worldwide reached an estimated 35 million.Chemotherapeutic agents were one of the most often implicated pharmacological classes in inducing adverse drug reactions. Adverse drug reactions increase the overall expense and mortality. Adverse drug reactions increase morbidity, mortality, hospitalization rate and financial expenses. Therefore, this study intended to assess chemotherapy-related adverse drug reactions and associated factors among adult cancer patients.

**Patients and method:**

A facility-based prospective observational study was conducted from July 2022 to October 2022 at Jimma Medical Center’s oncology unit. A standard data collection tool (Naranjo’s algorithm, modified Hartwig’s severity scale, and modified Schumock-Thornton criteria) was used for assessment of causality, severity, and preventability of adverse reactions, respectively. Socio-demographic profile and any adverse drug reactions reported were collected separately. The data was collected by one pharmacist and two nurses after giving training. Data was entered into Epidata version 4.6.0 and analyzed by SPSS version 25. Bivariate and multivariable logistic regression was conducted to identify independent predictors of the pattern of adverse drug reaction occurrence. A P-value of 0.05 was taken as statistically significant.

**Result:**

Out of 154 patients enrolled in the study, 66.2% were female. The mean age of patients was 41.20 ± 13.54 years. From the total, 98 (63.6%) cases developed a total of 198 adverse drug reactions. Out of them, 59.2% were female. The most commonly encountered adverse drug reactions were nausea and vomiting (33.8%) and hair loss (29.3%). Most of the reactions were probable (61.1%) in causality, mild (66.2%) in severity, and not preventable (43.9%) in nature. Female sex (AOR = 1.054; 95% CI= (1.021–1.087); P = 0.001), number of chemotherapy treatments (AOR = 3.33; 95% CI= (1.301–8.52); P = 0.012), and elderly age (AOR = 3.065; 95% CI= (1.01–9.296); P = 0.048) were associated with occurrences of adverse drug reactions.

**Conclusion:**

We can deduce from the data that adverse drug reactions are a significant concern for patients undergoing chemotherapy, with nearly two-thirds experiencing ADRs. The most common reactions are nausea and vomiting, which are mostly mild and probable. Age, gender, and the use of several chemotherapy drugs were associated with an increased risk of adverse drug reactions. Hence all concerned bodies should make an effort for early detection and take preventive measure of chemotherapy-related adverse drug reactions. Where feasible, use chemotherapy protocols with alower risk of ADRs. Evaluate dose adjustments for elderly patients. Implement protocols for risk assessment before initiating chemotherapy.

## Introduction

Adverse Drug Reaction (ADR) is defined as any reaction that is harmful and unplanned and happens at a dosage usually employed in humans for diagnosis, prophylaxis, and treatment of diseases or for a change in normal body function [1]. Chemotherapeutic agents are one of the common treatment modalities for cancer. Other modalities include radiotherapy, surgical operations, hormonal therapy, and biologic therapy [2–4]. Chemotherapy acts on all cells with little to no selectivity, which blocks the genetic material or the chemicals required for cell division. Normal body cells, like hair and skin cells, frequently divide. Chemotherapy has the potential to harm these cells as well, with negative effects, ADRs [5].

Adverse drug reactions (ADRs) have become a substantial global burden in healthcare and medicine, endangering patient safety. According to the Global Burden of Disease Study, ADRs commonly occur; the reported prevalence of ADRs is around 2.67 million and the incidence is near 35 million in 2017 [6]. Globally, during the last 10 years, 23 million ADRs were recorded in VigiBase, the World Health Organization’s Pharmacovigilance database. Antineoplastic drugs were one of the most frequently involved drug classes. On average, 68% of cancer patients develop chemotherapy-related ADRs in India [7]. Antineoplastic drugs are the most frequently involved drug classes in Egypt, which account for 43% of the total ADRs reported in a five years period [8]. In Ethiopia, the incidence of chemotherapy- related ADRs was around 50% [9,10].

Adverse drug reactions are one of the leading causes of mortality. During the last 10 years, a total of 230,000 patients died due to ADRs [11]. The prevalence of fatalities is around 0.11%. Antineoplastic agent-induced ADR (cytopenia) is one of the most common fatal drug-induced ADRs. The age of the patient and hospitalizations have an influence on the fatality rate [12]. Adverse drug reactions in affected patients carry a mortality risk of 10.7%, nearly 3-fold higher than those unaffected [13]. From total fatal ADRs in Egypt, 75% are due to chemotherapeutic agents. In Ethiopia, drug-related problems are substantial causes of mortality among patients presenting to emergency departments. Increments in drug-related problems may result from increasing access to complex treatment [14]. Fatal ADRs were common in hospitalized patients, and 1.5% of hospitalized patients died of ADRs in Ethiopia [15].

Adverse drug reactions account for 10% of hospital admissions, and 6% of hospital patients suffer from ADRs in the world [16]. In the United States, the incidence of ADR(s) in hospitalized patients accounted for 6.7% and was concluded to be the fourth to sixth leading cause of death in hospitalized patients [17]. Approximately 5% of total hospitalizations in Europe each year are due to ADR [18]. In Nepal, ADRs account for 3% to 6% of all hospital admissions, with 10% of hospitalized patients experiencing serious ADRs, and increase hospital stay by 1–6 days [19]. Thirty- six percent of cancer patients developing chemotherapy related ADRs stayed in the hospital for 1–4 days [20]. Up to 20 days increase in length of hospital stay in patients affected by chemotherapy- induced ADRs, which is twofold higher than those unaffected [13]. In Ethiopia, adverse drug reactions were a common cause of hospitalization, accounting for 10% of all hospitalizations [21] and about 12% of hospitalized patients developing at least one ADR [22].

Chemotherapy-related ADRs result in a substantial economic burden on patients, payers, caregivers, and society in general. Adverse drug reactions increase costs by increasing hospitalization, prolonging hospital stays, and requiring additional clinical investigations in more serious cases. In the USA, ADRs may cost up to 30.1 billion dollars annually [23]. Hematologic ADRs cost from 7100 to 37800 USD in the USA, costs of ADRs for patients with hematological malignancies appear 2–3 times higher than those for patients with solid tumors [24]. In India, chemotherapy induced ADRs may cost 457.23 USD direct medical costs per eight months [25]. In Nepal, chemotherapy induced ADRs increase an average of 33 USD per patient for additional hospital stay costs, clinical investigations of ADRs, and medications for management of ADRs [19]. ADRs pose a significant economic burden on the healthcare system by increasing hospital stays and treatment costs [22].

The most common ADRs associated with cancer chemotherapeutic agents are nausea, vomiting, diarrhea, and constipation; hematologic side effects such as neutropenia with infection, anemia, and bleeding; CNS side effects such as insomnia and headache; and other side effects such as anorexia, skin and nail discoloration, alopecia, weight loss, cough, fever, pain, and fatigue. An in-depth medication history is required to rule out any potential link between the presenting symptoms and an ADR and to identify the causative substance. Various criteria are used to diagnose an ADR, often in conjunction with precise objective investigation of the reaction [26–28].

A mix of hereditary and environmental variables causes the majority of adverse drug reactions. The extension of drugs intended pharmacologic effects is the primary cause of most adverse drug reactions (ADRs). Gender, age, weight, number of comorbidities, dosage, drug formulation, pharmacokinetic or pharmacodynamic qualities, administration method, number of concurrent medications, illness status, and prior ADR or allergy history are a few of the essential elements that frequently influence the development of ADRs. The most frequent causes of chemotherapy-induced ADR are alcohol use, educational level, treatment length and frequency, cancer kind and stage, and surgery [29–31].

Commonly used agents for ADR treatment are proton pump inhibitors, antiemetic tablets, iron tablets, and multivitamin and mineral tablets. Parenteral dexamethasone, ranitidine, ondansetron, and granisetron, as well as IV saline medications, are also used as supportive therapy to reduce chemotherapy-related ADRs. ADRs are also managed with the help of medications like hydrocortisone and pheniramine maleate [32,33]. Povidone-iodine gargle, chlorhexidine, and benzamine are other medications used for the prevention of chemotherapy induced ADRs. Besides medications, other prophylactic lifestyle modifications, such as maintaining good oral hygiene, avoiding spicy food, using mild-flavored toothpaste, were encouraged where appropriate for minimizing oral mucositis [19].

The pattern of chemotherapy-related ADRs in cancer patients in Ethiopia, specifically in our study setting, is not well studied. The safety profile of cancer chemotherapy in terms of causality, severity, and preventability in the study area is a scarcity of published information. By considering the narrow therapeutic index and high toxicity of these drugs, early recognition of drug toxicity helps modify the course of drug therapy to diminish toxic effects. We aim to describe the level problem of ADRs from chemotherapies; therefore, this study was to assess chemotherapy-related ADRs and associated factors among adult cancer patients at Jimma Medical Center, southwest Ethiopia.

## Patients and methods

### Study setting and period

The study was conducted at Jimma Medical Center (JMC) among patients with cancer attending the adult oncology unit from July 2022 to October 2022. JMC is the only tertiary referral center in southwest Ethiopia, with a catchment population of more than 20 million, 800 inpatient beds, and about 3000 hospital workers. It is located in Jimma Town, the biggest city in southwest Ethiopia, with a population of 300,000. It has 32 service units; the oncology center is one of the units. Jimma Medical Centre pediatric oncology unit was launched some five years ago, whereas the adult oncology unit was opened two years ago. The unit has received more than 2500 visitors as well as regular follow-ups for chemotherapy. Which is the only chemotherapy and radiotherapy center located in southwest Ethiopia.

### Study Design

Facility based prospective observational study was conducted at Jimma Medical center oncology unit.

### Population

#### Source population.

All adult cancer patients who were on chemotherapy visited JMC, oncology unit

#### Study population.

All adult cancer patients who were on chemotherapy visited JMC, oncology unit during the study periods and fulfill the eligibility criteria

### Inclusion and Exclusion criteria

#### Inclusion criteria.

- All adult cancer patients on chemotherapy who were willing to participate in the study
- All cancer patients who were on chemotherapy whose age > 18 years.

#### Exclusion criteria.

- Adult cancer Patients who develop ADR due to intentional overdose and poison

### Sample size and sampling technique

The sample size was calculated using a single population proportion formula using assuming the occurrence of adverse effect due to cancer chemotherapy is 45.5% [10] at 95% CI and a margin of error 5%and 10% non-response rate was considered.


$$The\;sample\;size\;n=Z({\frac{a}{2})}^{2}\frac{\;p(1-p)}{d^{2}}$$

Where

n = Sample size

α = level of significance

z = at 95% confidence interval Z value (α = 0.05) =>Z α/2 = 1.96

p = Assuming the occurrence of adverse effect due to cancer chemotherapy = 45.50%

d = Margin of error at (5%) (0.05)

n = ((${1.96\;}^{2}$ x 0.45(1-0.45))/ ${\left(0.05\right)}^{2}$

n = 380

- Since our source populations were less than 10,000, the sample size should be corrected using the following correction formula.

$n=\;\frac{no}{1+(\frac{no}{N})}$

$=\frac{380}{1+(\frac{380}{220})}$≈140

- Where **n** is the corrected sample size and N is the number of patients visiting the hospital in the previous three months, which was 220.

10% non-response rate=14, so the total sample size (n) was

n = 140+ 14 = 154

A consecutive sampling technique was used to recruit the study participants until the final sample size reach.

### Data collection instrument and processing

The data regarding ADRs and patient details was directly collected from patients and their case files and/or medical charts using a semi-structured questioner. Socio-demographic and clinical details of the patients and details of the medications given were carefully recorded initially. The occurrence and nature of ADR and any relevant laboratory investigation values were collected during every chemotherapy cycle visit within the three month study period. All the study participants were assessed for ADRs at every chemotherapy cycle visit with in the study period with a minimum of one chemotherapy cycle. The information about adverse events during the prior cycle was collected at the beginning of the next cycle, along with some data from the last collected cycle. The reported ADR was assessed for causality using Naranjo’s algorithm. The Naranjo’s Algorithm, a questionnaire designed by Naranjo’s et al, [34] consists of 10 questions with three types of responses: yes, no, or do not know. Scores are given accordingly, and the drug reaction can be classified as definite, probable, possible, and doubtful based on the total score. The severity of ADR’s was assessed using the modified Hart wig’s severity scale [35]. It classifies the severity of ADR as mild, moderate, or severe with various levels according to factors such as requirements for change in treatment, duration of hospital stay, and disability produced by the ADR. The preventability of ADRs was assessed using modified Schumock–Thornton criteria [36]. It consists of 9 sequential questions with yes/no responses. It classifies the preventability of ADRs as definitely preventable, probably preventable, and not preventable. Answering “yes” to one or more of the first three questions implies that the ADR is definitely preventable. If answers are all negative to the first three questions, then proceed to Section B. Answering “yes” to one or more of the questions in Section B implies that the ADR is probably preventable. If answers are all negative to the questions in sections A and B, then proceed to section C. The ADR could not have been avoided by any reasonable means, implies that the ADR is not preventable.

### Data quality assurance

The semi-structured questionnaire and standardized tools prepared in English were used. A pre-test was conducted on 5% of study participants by randomly selected patients before the actual data collection to check the consistency and validity of the data collection format. Training was given for one pharmacist and two nurses for a couple of days regarding the aim of the study, eligibility criteria, and sampling technique before data collection. The information obtained from each consented patient was filled out by one pharmacist and two oncology nurses at the Jimma Medical Center oncology unit. All collected data was checked for completeness by the principal investigator on a daily basis, and corrective actions were sent back to data collectors, if any. To ensure the accuracy of data, the double data entry method was used.

### Data processing and satastical analysis

The collected data was checked for its completeness and coded. And then it was entered into Epidata version 4.6.0 and exported to SPSS version 25 software to analyze, manage, and produce graphical visualizations of data. The continuous variable was reported by mean with standard deviation, and the categorical variable was summarized using percentages and frequency tables. Bivariate and multivariable logistic regression models were used to assess the factors associated with the pattern of chemotherapy-related ADR occurrence. Variables having a p-value of ≤ 0.25 in the bivariate analysis were subjected to multivariable analysis to identify independent predictors of chemotherapy-related ADRs. p-value<0.05 was considered statistically significant.

### Ethics approval and consent to the participants

Ethical approval and clearance were obtained from the Jimma University Institutional Review Board, with the ethical approval number of IHRPGY/7215/2022. A supportive letter was written to Jimma Medical Center oncology unit medical directors or managers. Data collection was started after obtaining permission from hospital managers or medical directors and written informed consent from the study participants or caregivers. The study subjects names or identities were not mentioned on the request paper, and their privacy was kept confidential.

### Definition of terms

Adverse drug reactions: an appreciably harmful or unpleasant reaction resulting from an intervention related to the use of medicinal products [37].

The Naranjo’s Scale total scores range from -4 to + 13; the reaction is considered;

Definite: causative if the Naranjo’s score is 9 or higher: The reaction followed a reasonable temporal sequence after a drug or in which a toxic drug level had been established in body fluids or tissues, followed a recognized response to the suspected drug and was confirmed by improvement on withdrawing the drug and reappeared on re-exposure.

Probable: if the Naranjo’s score is 5–8: The reaction followed a reasonable temporal sequence after a drug, followed a recognized response to the suspected drug, was confirmed by withdrawal but not by exposure to the drug, and could not be reasonably explained by the known characteristics of the patient’s clinical state.

Possible: if the Naranjo’s score is range from 1to 4: The reaction followed a temporal sequence after a drug, possibly followed a recognized pattern to the suspected drug and could be explained by characteristics of the patient’s disease.

Doubtful: if the Naranjo’s score is 0 or less: The reaction was likely related to factors other than a drug.

According to Hart wig’s ADR severity scale;

Mild: An ADR occurred required or no to held, discontinued, or otherwise changed to the suspected drug. No antidote or other treatment requirement was required and no increase in length of stay.

Moderate: The mild ADR and/or an Antidote or other treatment was required. May or no increase in length of stay by at least 1 day or the ADR was the reason for the admission.

Severe: Any moderate ADR which requires intensive medical care or caused permanent harm or directly or indirectly led to the death of the patient.

According to The Schumock and Thornton ADRs preventability criteria;

Definitely preventable: If patients have history of allergy or previous reactions to the drug or the drug involved Was inappropriate for the patient’s clinical condition or the dose, frequency, or route of administration Was inappropriate for the patient’s age, weight, or disease status.

Probably preventable: if required therapeutic drug monitoring or other necessary laboratory tests was not done or documented drug interaction was involved in the ADR or the patients have poor compliance or if preventive measure was not administered/it was inadequate and/or inappropriate to the patient.

Non-preventable: if the ADR could not have been avoided by any reasonable means.

## Results

**Fig 1:**
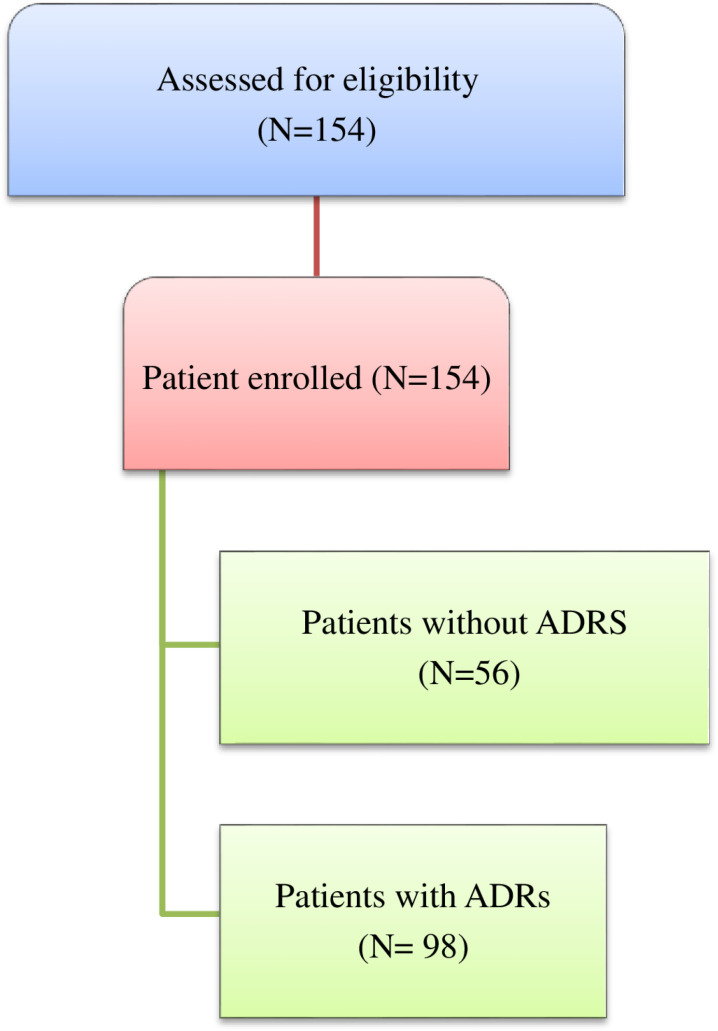
Study participant enrollment at Jimma Medical Center from July 2022 to October 2022.

One hundred fifty-four patients with cancer received treatments of chemotherapy only or in combination with other treatment modalities during the period of study. All the patients fulfilled the inclusion criteria, so no patients were excluded from the study. (Fig 1)

### Socio-demographic and clinical data

From the total 154 patients who received chemotherapy during the study period, around two-thirds of the study participants (66.2%) were females. The mean age of patients was 41.20 ± 13.54 years, the mean weight of the study participants was 54.4279 ± 12.0315, and the mean body surface area of the study participants was 1.5376 ± 0.17752. Other socio-demographic characteristics are presented below. (Table 1)

**Table 1:** Socio-demographic characteristic of cancer patients, JMC, Ethiopia (N = 154).

Variables	Categories	Frequency (%)
Sex	Female	102(66.2)
	Male	52(33.8)
Age	18-30	46(29.9)
	31-40	33(21.4)
	41-50	29(18.8)
	51-60	11(7.1)
	≥61	36(23.4)
Marital status	Married	131(85.1)
	Widowed	14(9.1)
	Divorced	5(3.2)
	Unmarried	4(2.6)
Alcohol status	No	148(96.1)
	Yes	6(3.9)
Smoking status	No	152(98.7)
	ex-smoker	2(1.3)
Educational level	Can’t read and write	58(37.7)
	Primary school	53(34.4)
	Secondary school	24(15.6)
	College and university	19 (12.3)
Occupational status	house wife	65(42.2)
	Farmer	40(26.0)
	civil servant	28(18.2)
	Businessmen	18(11.7)
	Student	3(1.9)

### Patients clinical characteristics

**Fig 2:**
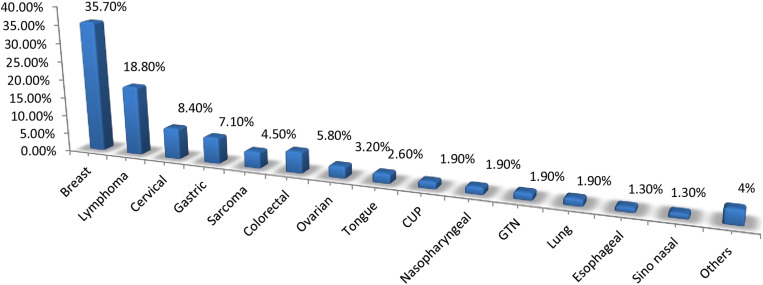
Types of Tumor diagnosed, JMC, Ethiopia (N = **154).**

**Fig 3:**
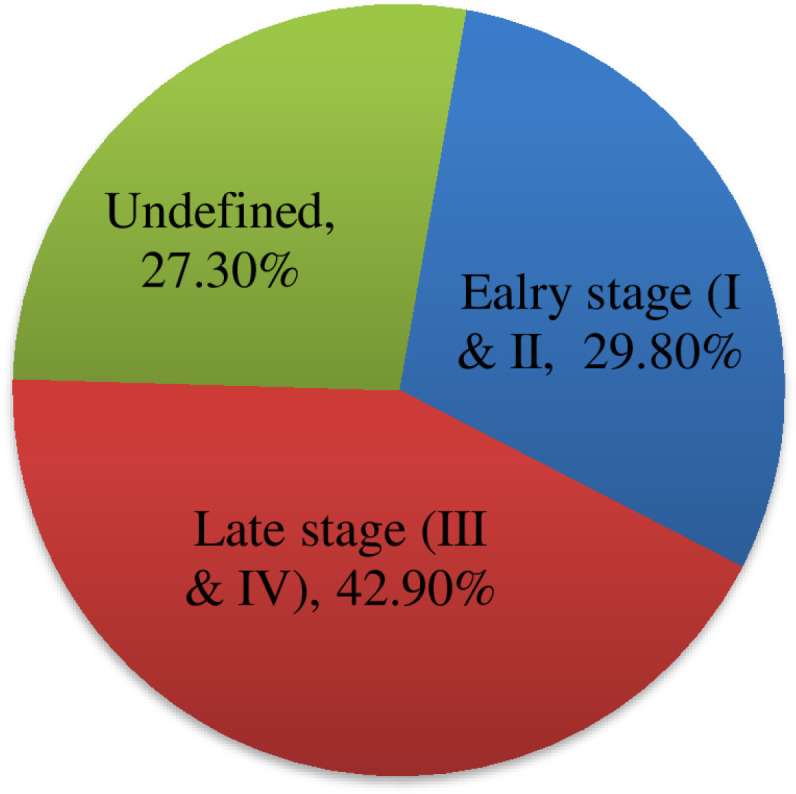
Stages of diagnosed cancer, JMC, Ethiopia (N = **154).**

The most common types of cancer diagnosed in the study participants were breast cancer (35.7%), followed by hematologic malignancies (29 (18.8%); 21 non-Hodgkin’s lymphoma; 6 Hodgkin’s lymphoma); gastrointestinal malignancies (22 (14.3%); 11 gastric, 7 colorectal), and gynecologic malignancies (18 (11.7%); 13 cervical, and 5 ovarian cancers. (Fig 2) Most of the diagnosed cancers were categorized under late stage, which is stage III and IV 66 (42.9%). (Fig 3) Comorbid medical conditions were present in 6.5% of the study participants (the most common comorbid condition diagnosed was hypertension). (Table 2)

**Table 2:** Comorbid conditions identified in the study participant’s, JMC, Ethiopia (N = 154).

Comorbidity	Frequency (%)
Hypertension	6(3.9)
RVI	2(1.3)
Diabetes	1(0.6)
Diabetes +Hypertension	1(0.6)

Abbreviation; JMC, jimma medical center; RVI, retroviral infection.

### Treatment characteristics

The majority of research subjects, 57 (37.0%), received chemotherapy and surgery. Two chemotherapeutic drugs were administered to over half of the patients (57.1%; 88). From the study participants 49 (31.7%) received treatment for more than four cycles. (Table 3)

**Table 3:** Treatment characteristics of cancer patients, JMC, Ethiopia (N = 154).

Treatment modality	Frequency (%)
Chemotherapy only	81 (52.6)
Chemotherapy and surgery	57 (37.0)
Chemotherapy and radiation	14 (9.1)
Chemotherapy, radiation and surgery	2 (1.3)
Number of Chemotherapy agents	
Monochemotherapy	21 (13.6)
Dichemotherapy	81 (52.6)
Polychemotherapy	52 (33.8)
Number of Chemotherapy cycles	
1^st^ cycle	27 (17.5)
2^nd^ cycle	21 (13.6)
3^rd^ cycle	18 (11.7)
4^th^ cycle	39 (25.3)
>4^th^ cycle	49 (31.7)

### Patterns of adverse drug reactions

#### Types of adverse drug reactions.

Of 154 patients who received chemotherapy during the study period a total of 198 ADRs with an average of 2.02 ADRs per patient. More than half of the patients (59.1%) were females. The most commonly occurring ADRs were nausea and vomiting (33.8%), followed by hair loss (29.3%), loss of appetite (12.6%), diarrhea (11.1%), febrile neutropenia (3.5%), and constipation (3.0%) (Table 4).

**Table 4:** Types of adverse drug reactions identified, JMC, Ethiopia (N = 154).

ADRs	Frequency (%)
Nausea and vomiting	67(33.8)
Hair loss	58(29.3)
Loss of appetite	25(12.6)
Diarrhea	22(11.1)
Febrile neutropenia	7(3.5)
Constipation	6(3.0)
Anemia	5(2.5)
Mucositis	4(2.0)
Nail discoloration	2(1.0)
Bell’s palsy	1(0.5)
Thrombocytopenia	1(0.5)
Total	198

#### Suspected chemotherapeutic agents causing ADRs.

**Fig 4:**
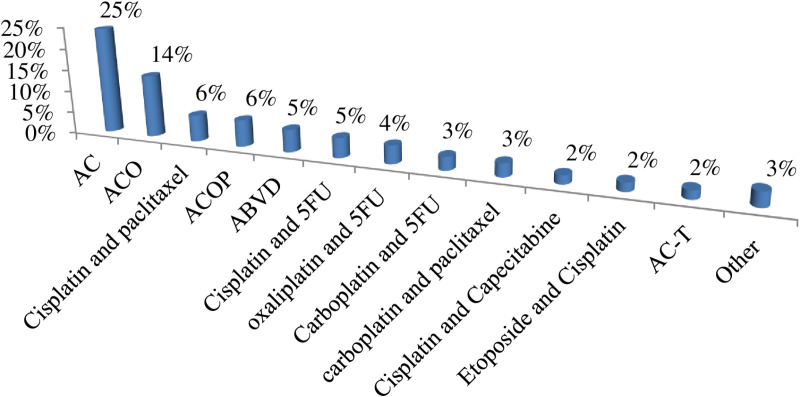
Suspected combined chemotherapeutic agent causing ADRs, JMC, Ethiopia (N = **154).**

**Fig 5:**
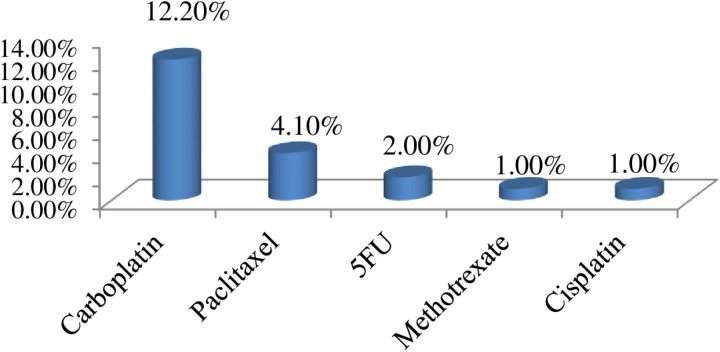
Suspected single chemotherapeutic agents causing ADRs, JMC, Ethiopia (N = **154).**

The most common chemotherapeutic agents used in our study participants were doxorubicin, cyclophosphamide, vincristine, paclitaxel, and 5-FU in a combination regimen for the treatment of the common cancers in the setting, such as breast cancer and lymphomas. Carboplatin, cisplatin, oxaliplatin, bleomycin, decarbazine, and prednisolone were the other drugs used. The most common drugs causing ADRs were doxorubicin, cyclophosphamide, vincristine, and carboplatin, causing more than 50% of ADRs in combination (AC 25%, ACO 14%). (Fig 4) The most common single chemotherapeutic agent causing ADRs was carboplatin 12%. (Fig 5)

#### Causality assessment of suspected drugs and ADRs.

Assessment of causality by Naranjo’s algorithm: from the total 198 ADRS, 61.1% of the reactions were “probable,” with a score ranging from 5 to 8, and 19.7% were “possible,” with a score ranging from 1 to 4 (Table 5).

**Table 5:** The Causality assessment scales, JMC, Ethiopia (N = 154).

ADRs	Number of adverse drug reactions Naranjo’s algorithm				Total (%)
	Definite, N (%)	Probable, N (%)	Possible, N (%)	Doubtful, N (%)	
Nausea and vomiting	19 (28.4)	36 (53.7)	11(16.4)	1(1.5)	67(33.8)
Hair loss	–	53(91.4)	5(8.6)	–	58(29.3)
Febrile neutropenia	4(57)	3(43)	–	–	7(3.5)
Anemia	1(20)	3(60)	1(20)	–	5(2.5)
Constipation	–	3(50)	3(50)	–	6(3.0)
Diarrhea	7(31.8)	8(36.4)	5(22.7)	2(9)	22(11.1)
Mucositis	1(25)	3(75)	–	–	4(2.0)
Loss of appetite	3(12)	9(36)	13(52)	–	25(12.6)
Nail discoloration	–	2	–	–	2(1.0)
bell’s palsy	–	–	1	–	1(0.5)
Thrombocytopenia	–	1	–	–	1(0.5)
Total	35(17.7)	121(61.1)	39(19.7)	3(1.5)	198(100)

#### Severity assessment of the identified ADRs.

The severity of the reported reactions was graded by using Hartwig’s severity scale. Accordingly, of the identified 198 ADRs, 66.2% were mild and 33.3% were moderate. The most common mild toxicities were hair loss (100%) and loss of appetite (72%). (Table 6)

**Table 6:** Severity assessment scales, JMC, Ethiopia (N = 154).

ADRs	Number of adverse drug reactions Hartwig’s algorithm (%)			Total N (%)
	Mild N (%)	Moderate N (%)	Severe N (%)	
Nausea and vomiting	30(38.8)	37(55.2)	–	67(33.8)
Hair loss	58(100)	–	–	58(29.3)
Febrile neutropenia	4(57.1)	3(42.9)	–	7(3.5)
Anemia	2(40.0)	3(60.0)	–	5(2.5)
Constipation	6(100.0)	–	–	6(3.0)
Diarrhea	8(36.4)	14(63.6)	–	22(11.1)
Mucositis	3(75.0)	1(25.0)	–	4(2.0)
Loss of appetite	18(72.0)	7(28.0)	–	25(12.6)
Nail discoloration	2	–	–	2(1.0)
bell’s palsy	–	–	1	1(0.5)
Thrombocytopenia	–	1	–	1(0.5)
Total	131(66.2)	66(33.3)	1(0.5)	198(100.0)

#### Preventability assessment of the identified ADRs.

Assessment of the preventability showed that from the total 198 ADRs, 56% were preventable (31.8% probably preventable, 24.3% definitely preventable) in nature, and the rest 43.9% of the ADRs were not preventable (Table 7).

**Table 7:** Preventability assessment scales, JMC, Ethiopia (N = 154).

ADRs	The Schumock and Thornton scale preventability algorithm			
	Definitely preventable, N (%)	Probably preventable, N (%)	Not preventable, N (%)	Total
Nausea and vomiting	28(41.8)	20(29.9)	19(28.3)	67
Hair loss	–	–	58(100)	58
Febrile neutropenia	3(42.9)	4(57.1)	–	7
Anemia	1(20)	1(20)	3(60)	5
Constipation	1(16.7)	5(83.3)	–	6
Diarrhea	8(36.4)	10(45.4)	4(18.2)	22
Mucositis	1(25)	3(75)	–	4
Loss of appetite	6(24)	19(76)	–	25
Nail discoloration	–	–	2	2
bell’s palsy	–	–	1	1
Thrombocytopenia	–	1	–	1
Total	48 (24.3)	63 (31.8)	87(43.9)	198

#### Factors associated with the occurrence of ADRs.

The following variables were tested for their association with ADRs: sex, age, educational level, occupation, smoking status, alcohol use, type of cancer, stage of cancer, presence of co-morbidity, treatment modality, number of chemotherapies, number of chemotherapy cycles, and concurrent drug use.

In the bivariate logistic regression analysis, it was illustrated that the predictor of ADR occurrences was more likely associated with female sex. COR = 3.604, CI = 1.668–7.788, P = 0.001, Age of the patients: COR = 4.166, CI = 1.63–10.645, p = 0.003. Comorbidity COR = 1.056, CI = .764-3.406, p = 0.001, Number of chemotherapy COR = 3.139, CI = 1.494–6.596, P = 0.003, Number of chemotherapy cycles COR = 1.569, CI = .756-3.255, P = 0.009. All variables with a p-value <0.25 on binary logistic regression were entered into a multivariable logistic regression to control for confounding.

From the above bivariate output, patient sex, patient age, and number of chemotherapys are found to be independent predictors of ADR occurrences in multivariate logistic regression analysis. Female cancer patients were 1.054 times (AOR = 1.054; 95% CI= (1.021–1.087); P = 0.001) more likely to develop ADRs as compared to male cancer patients. Cancer patients whose age greater than 60 were 3.065 times (AOR = 3.065; 95% CI= (1.01–9.296); P = 0.048) more likely to develop ADRS as compared to patients whose age less than 30. Cancer patients who took four chemotherapeutic agents were 3.33 times (AOR = 3.33; 95% CI = 1.301–8.52); P = 0.012) more likely to develop ADRs compared with those patients who took only one chemotherapeutic agent. In general, patients’s sex being female, elderly patients greater than 60 years, and numbers of chemotherapeutic agents greater than four were positively associated with the occurrence of chemotherapy-induced ADRs. (Table 8)

**Table 8:** Factors associated with the occurrence of ADRs, JMC, Ethiopia (N = 154).

Variables	Categories	ADRs		COR(95%CI)	P-value	AOR(95% CI)	P-value
		Yes N (%)	No N (%)				
Sex	Male	40(58)	29(42)		1		1
	Female	58(61.1)	37(39)	3.604(1.668-7.788)	0.001	1.054(1.021-1.087)	0.001
Age	18-30	20(43.5)	26(56.5)		1		1
	31-40	19(57.6)	14(42.4)	1.56(.786-3.023)	0.206	0.085(.507-5.613)	0.29
	41-50	18(62)	11(38)	1.00(.563-4.515)	0.117	1.014(.071-12.186)	0.8
	51-60	6(54.5)	5(45.5)	1.400(.946-10.383)	0.015	1.024(.794-4.646)	0.17
	≥61	31(86)	5(14)	4.166(1.63-10.645)	0.003	3.065(1.01-9.296)	0.048
Comorbidity	Yes	7(54.5)	4(45.5)	1.056(.764-3.406)	0.001	1.027(.794-4.646)	0.981
	No	88(61.1)	56(38.9)		1		1
No of chemotherapy	1	11(52.4)	10(47.6)		1		1
	2	44(51.8)	37(48.2)	1.542(.786-3.023)	0.207	1.673(.74-3.782)	0.216
	3	22(81.5)	5(18.5)	1.629(.616-4.306)	0.326	1.206(.4-3.636)	0.74
	4	20(80)	5(20)	3.139(1.494-6.596)	0.003	3.33(1.301-8.52)	0.012
No of chemo cycle	1	23(85.2)	4(14.8)	1.569(.756-3.255)	0.009	1.427(.531-3.834)	0.927
	2	16(76.2)	5(23.8)	1.492(.762-2.92)	0.998	1.673(.667-4.197)	0.289
	3	12(66.7)	6(33.3)	1.843(.805-4.223)	0.147	1.08(.298-3.918)	0.16
	≥4	39(43.3)	49(55.7)		1		1

Abbreviations; COR=crude odd ratio, AOR=adjusted odd ratio, CI=confidence interval, ADRs=adverse drug reactions, JMC=jimma medical center

## Discussion

This pioneer study in the study setting shows chemotherapy-induced ADR occurrence, causality, severity, and preventability. After the data were collected and analyzed, we observed that 63.6% (CI: 56.2–70.4%) of the study populations develop ADRs during cancer chemotherapy, which is comparable with 59 and 58.6% that were reported in two studies conducted in India [38,39]. Whereas a study conducted in Ethiopia showed a lower prevalence (45.5%) of ADR [10]. This difference may be due to different medications and a different study design used in the study.

The incidence of ADRs was greater in female participants (59.1%) as compared with male participants (40.9%), which is supported by research reported from India and Nepal [40,41]. However, other studies from different parts of India showed a higher occurrence of ADRs in male patients [38,42,43]. The difference can be explained by the higher incidence of breast cancer (35.7%) seen in our study setting.

Doxorubicin, cyclophosphamide, vincristine, Cisplatin, paclitaxel, and 5-FU-containing regimens, as well as carboplatin as a single agent, were the common agents found to be associated with most of the ADRs in our setting, which is supported by two studies reported in Ethiopia and India [9,40].

The system that was most frequently affected by ADRs was the gastrointestinal tract (GIT), which is supported by studies reported in India and China [39,44]. A total of 33.8% (CI: 24.5–43.2%) cases of nausea and vomiting were reported in our setting, which is comparable with 25.5% that was reported in a study conducted in central India [42]. Whereas a study done in north-eastern indial show lower prevalence of nausea and vomiting [39].The most common mechanism of the ADR is the activation of the chemoreceptor trigger zone.

Alopecia (29.3%) (CI: 20.3–38.3%), the second most common ADR, is compared with 20.6%, 20.75%, and 20.9% that were reported in other studies reported in different parts of India [38,39,42].

The next most common ADR was found to be loss of appetite (12.6%) (CI: 5.1–17.7%), compared with 9% reported in a study done in Ethiopia [45]. In our study, a total of 11.1% (CI:3.05–14.15%) of patients suffered from diarrhea, which is compared with 7.81%, 7.1%, and 6.3% that were reported in three studies done in different parts of India (12,28,33).

On analyzing the causality assessment of the ADRs by Naranjo’s score, we found that 61.1% (CI: 51.4–70.8%) of cases showed probable association and 19.7% showed possible association, which is comparable with studies done in central India, South India, and Nepal, which reported 61%, 65%, and 66% of probable scores using the same scale [19,40,42]. Whereas another study done in north-eastern India reported that 80% of the ADRs were probable in the causality score [38].

Analyses of the severity of these adverse reactions using Hartwig’s severity scale showed that the majority of the ADRs (66.2%) were mild, followed by moderate (33%). This finding was comparable with studies done in northeastern and southern India, where the maximum number of ADRs were in the mild category [38,46]. Whereas the study done in Nepal reported that 73.4% of the ADRs were moderate, while only 20.5% were mild [19]. The difference seen may be due to the age difference of the study participants involved; the majority of the participants in that study were ages greater than 60 years.

Assessment of preventability by the Schumock and Thornton scale showed that only 34.3% of the ADRs categorized under probably prevented were in patients who had symptoms of vomiting and loss of appetite when appropriate premedication was given before the start of chemotherapy. In our study, most (43.9%) of the recorded ADRs were not preventable. This result is supported by two studies done in India, where an average of 47.3% of ADRs were categorized under the not preventable category [38,39].

The present study revealed that female cancer patients were 1.054 times more likely to develop chemotherapy-induced ADRs as compared to male cancer patients. This study’s finding is supported by a study done in the United States that reported that female cancer patients were 2.79 times more likely to develop chemotherapy-induced ADRs as compared to male cancer patients [30]. This may be explained by females have higher fat-to- lean bodymass ratios, hormonal fluctuations, increased sensitivity, and exhibit slower drug clearance than males. Whereas a study done in India reported that male participants were significantly associated with the development of ADRs [47].

In our study, cancer patients whose ages were greater than 60 were 3.065 times more likely to develop chemotherapy-induced ADRs as compared to patients whose ages were less than 30. Which is supported by studies conducted in India and Japan that showed that older patients were 2.22 times and 2.03 times more likely to develop chemotherapy-induced ADRs, respectively [39,48]. Another study done in Ethiopia also reported that age greater than 65 was significantly associated with the occurrence of chemotherapy-induced ADRs [9]. This may be due to elderly patients have age related physiologic changes, multiple comorbidity, and impaired homeostasis than younger individuals. In contrast with our findings, a study conducted in the United States showed that younger cancer patients were 2.59 times more likely to develop chemotherapy-related ADRs as compared to older cancer patients [30].

The present study also revealed that cancer patients who took four chemotherapeutic agents were 3.33 times more likely to develop chemotherapy-related ADRs compared with those who took only one chemotherapeutic agent. A study done in northeastern Ethiopia supports our finding that reported that patients taking four or more chemotherapy agents developed 2.67 times more ADRs than patients who took a single chemotherapeutic agent [45]. Another study conducted in Ethiopia reported that cancer patients who had taken multiple medications had 1.4 times the risk of developing chemotherapy-induced ADRs as compared to patients who took only one agent [10]. This may be explained by polypharmacy carries multiple drug interactions,additive and cumulative drug toxicities than single agent. In contrast to our findings, one study done in Ireland reported that age, gender, and medication number had no association with the occurrence of chemotherapy-related ADRs [26].

## Limitations

One of the study’s drawbacks was that we only looked at three months’ worth of data, which limited the number of study participants and the amount of data we could collect, which directly affecets the analysis and interpretation of our study. Other limitations included the absence of comprehensive information about supportive care therapies and every patient was not evaluated for the same number of visits. The present investigation is limited by the fact that it was conducted exclusively in one cancer center.

## Conclusion

We can deduce from the data that adverse drug reactions are a significant concern for patients undergoing chemotherapy with nearly two-thirds experiencing ADRs. The most common reactions are nausea and vomiting, which are mostly mild and probable. Age, gender, and the use of several chemotherapy drugs were associated with an increased risk of adverse drug reactions. Hence all concerned bodies should make an effort for early detection and take preventive measure of chemotherapy-related adverse drug reactions. Where feasible, use chemotherapy protocols with alower risk of ADRs. Evaluate dose adjustments for elderly patients. Implement protocols for risk assessment before initiating chemotherapy.

## Supporting information

S1 Data Chemo ADRs datas. (XLSX)

## Abbreviation/Acronym:

ADR: adverse drug reactions
CT: chemotherapy
CINV: chemotherapy induced nausea and vomiting
DRPs: drug related problems
JMC: jimma medical center
SPSS: statistical package for social sciences
WHO: World Health Organization
